# Irisin inhibits pancreatic cancer cell growth via the AMPK-mTOR pathway

**DOI:** 10.1038/s41598-018-33229-w

**Published:** 2018-10-15

**Authors:** Jiayu Liu, Nannan Song, Yibing Huang, Yuxin Chen

**Affiliations:** 1 Key Laboratory for Molecular Enzymology and Engineering of the Ministry of Education, Jilin University, 2699 Qianjin Street, Changchun, 130012 China; 2 School of Life Sciences, Jilin University, Changchun, 130012 China

## Abstract

Irisin, a recently identified myokine that is released from skeletal muscle following exercise, regulates body weight and influences various metabolic diseases such as obesity and diabetes. In this study, human recombinant nonglycosylated P-irisin (expressed in *Escherichia coli* prokaryote cell system) or glycosylated E-irisin (expressed in *Pichia pastoris eukaryote* cell *system*) were compared to examine the role of recombinant irisin against pancreatic cancer (PC) cells lines, MIA PaCa-2 and Panc03.27. MTT [3-(4, 5-dimethylthiazol-2-yl)-2, 5-di phenyltetrazolium bromide] and cell colony formation assays revealed that irisin significantly inhibited the growth of MIA PaCa-2 and Panc03.27 in a dose-dependent manner. Irisin also induced G1 arrest in both cell lines. Scratch wound healing and transwell assays revealed that irisin also inhibited the migration of PC cells. Irisin reversed the activity of epithelial–mesenchymal transition (EMT) while increasing E-cadherin expression and reducing vimentin expression. Irisin activated the adenosine monophosphate-activated protein kinase (AMPK) pathway and suppressed the mammalian target of rapamycin (mTOR) signaling. Besides, our results suggest that irisin receptors exist on the surface of human MIA PaCa-2 and Panc03.27 cells. Our results clearly demonstrate that irisin suppressed PC cell growth via the activation of AMPK, thereby downregulating the mTOR pathway and inhibiting EMT of PC cells.

## Introduction

Pancreatic cancer (PC) is a malignant tumor of the digestive system, which involves the formation of malignant cells in pancreatic tissues^1,2^. It is a highly lethal disease that is often difficult to diagnose until later stages. Only few effective treatments are available for PC^1^. According to the data from the American Cancer Society, the 5-year survival rate of pancreatic ductal adenocarcinoma is only 7% for all stages, and the incidence rate of PC is yearly increasing worldwidely^3^. The cause of PC is not well established^4^. In recent years, progressive research evidence has indicated the association of PC with obesity, insulin resistance, and diabetes mellitus^5,6^.

Irisin has been recently identified as a myokine secreted from skeletal muscle^7^ and ubiquitously present in testis, pancreas, liver, spleen, brain, stomach, adipose tissues, skin tissues, and heart^8^. Studies have demonstrated that irisin is released upon cleavage of the membrane of fibronectin type III domain-containing protein 5 (FNDC5) and is increased following exercise^7^. Irisin has been reported to induce browning of white adipose tissues by upregulating the peroxisome proliferator-activated receptor (PPAR)-γco-activator 1α (PGC-1α) and uncoupling protein 1 (UCP1)^7^. It has been reported that irisin can potentially act as a therapeutic agent in the management of obesity and several metabolic diseases^9–11^. It is generally known that obesity is a risk factor for various cancers^6,12^. Recently, several researchers have focused their attention on the relationship between irisin and cancer. According to recent studies, irisin exhibits suppressive effects on the number and migratory characteristics of malignant breast cancer cells^13^, inhibits the proliferation, EMT, and the activation of the PI3K/AKT pathway in lung cancer cells^14^. In addition, irisin inhibits the proliferation, migration, and invasion of osteosarcoma cells and reverses the IL-6 induced EMT in osteosarcoma cells via the STAT3–Snail signaling pathway^15^. In contrast, irisin has been reported to stimulate cell proliferation and invasion by targeting the PI3K/AKT pathway in human hepatocellular carcinoma^16^. Studies have revealed significantly increased irisin levels in nearly all gastrointestinal cancers, including gastric adenosquamous carcinoma, esophageal adenocarcinoma, and colon adenocarcinoma, but not in liver cancers. Immunohistochemical studies have revealed that irisin is significantly increased in the pancreatic intra- and interlobular ducts of ductal adenocarcinoma than in normal pancreas^17^. Boström *et al*. have proposed that irisin acts by binding to unidentified receptors^7^. Few reports have suggested that irisin receptors reside in the membrane of cardiomyoblasts^18^ and preadipocytes^19^. Based on the results of these studies, we consider that irisin affects PC. In addition, biochemical data have confirmed that irisin is a dimer and that dimerization is unaffected by glycosylation^20^. To verify whether glycosylation affects the antitumor activity of irisin, we expressed and purified human recombinant non-glycosylated P-irisin in *Escherichia coli* and human recombinant glycosylated E-irisin in *Pichia pastoris*. In this study, we evaluated the influence of irisin on PC cell growth, migration, invasion, and EMT and explored the underlying mechanisms. Furthermore, irisin receptors in cancer cells were also investigated.

## Results

### Irisin receptors may exist on the surfaces of PC cells

**Figure 1:**
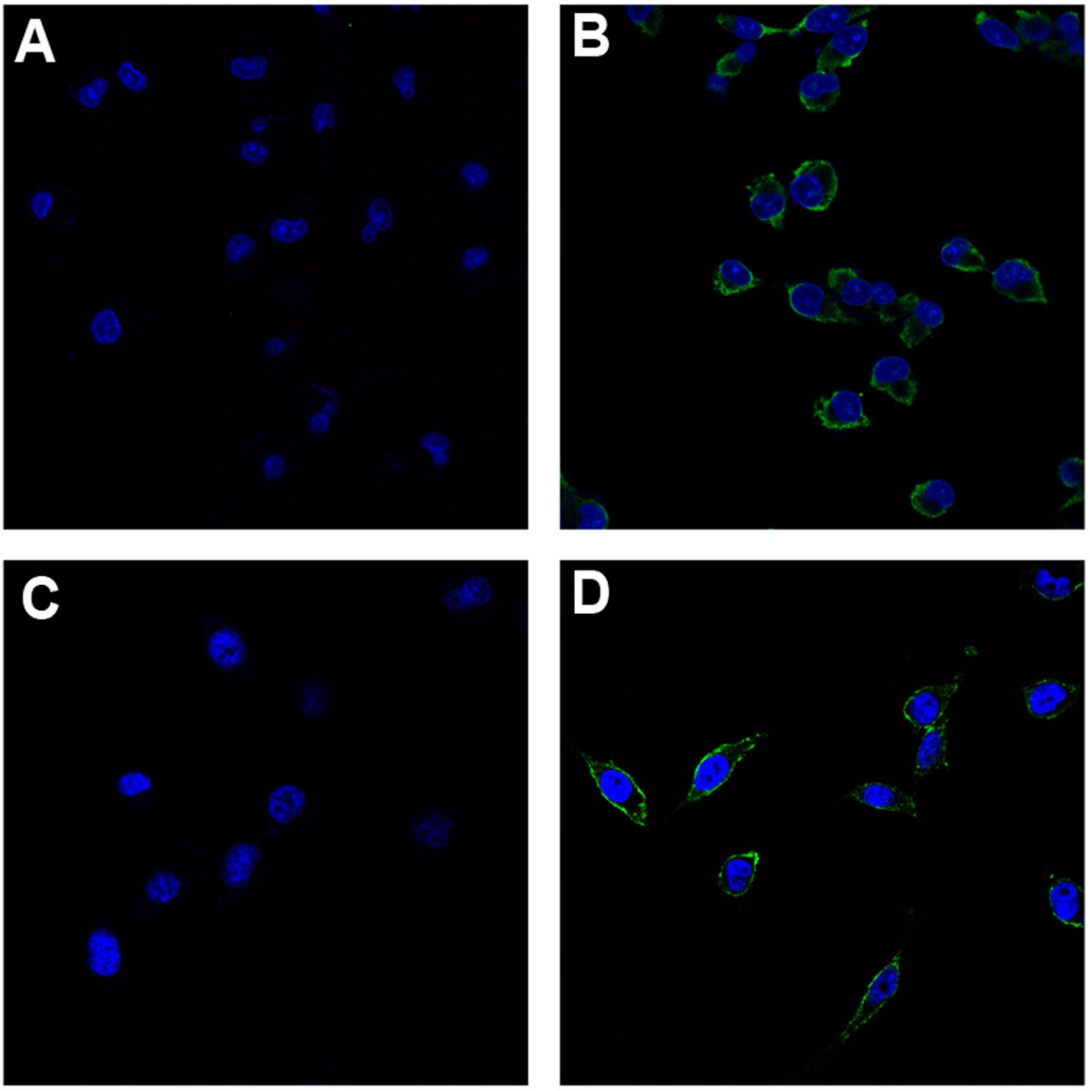
Immunofluorescence detection of irisin receptors on pancreatic cancer cell membrane. Images were taken using the confocal fluorescence microscope. MIA PaCa-2 (**A**,**B**) and Panc03.27 (**C**,**D**) cells were previously treated with or without irisin and incubated with anti-irisin rabbit antibody and fluorescein FITC (green) conjugated secondary antibody. Cell nuclei were stained with Honchest 33258 (blue). (**A**,**C**) Immunofluorescence staining of control cells that were treated without irisin. Green fluorescence was not observed on the cell membrane of control cells. (**B**,**D**) Visible green fluorescence was observed on the membrane of cells pretreated with irisin. The presence of green fluorescence indicates the presence of irisin receptors on the membranes of MIA PaCa-2 and Panc03.27 cells.

Immunofluorescent staining of MIA PaCa-2 and Panc03.27 cells was performed to detect the binding of irisin to the membrane of PC cells. Cells in positive groups were previously treated with irisin, incubated with anti-irisin rabbit antibody (Human specific), and then treated with FITC-anti-rabbit IgG antibody (FITC could emit bright green fluorescence by confocal laser scanning microscope). Cells treated without irisin, but still with anti-irisin rabbit antibody, and FITC anti-rabbit IgG antibody served as negative controls. We believed that there may be receptors on the cell membrane that irisin could bind to. Irisin binding to the cell membrane potentially could be detected by immunofluorescence. As anticipated, only cells to which irisin was added in advance showed green visible fluorescence on its membrane (Fig. 1) compared to the negative controls. These results suggest that MIA PaCa-2 and Panc03.27 PC cell lines have irisin receptors on their cell membrane.

### Irisin inhibits the growth of PC cells

**Figure 2:**
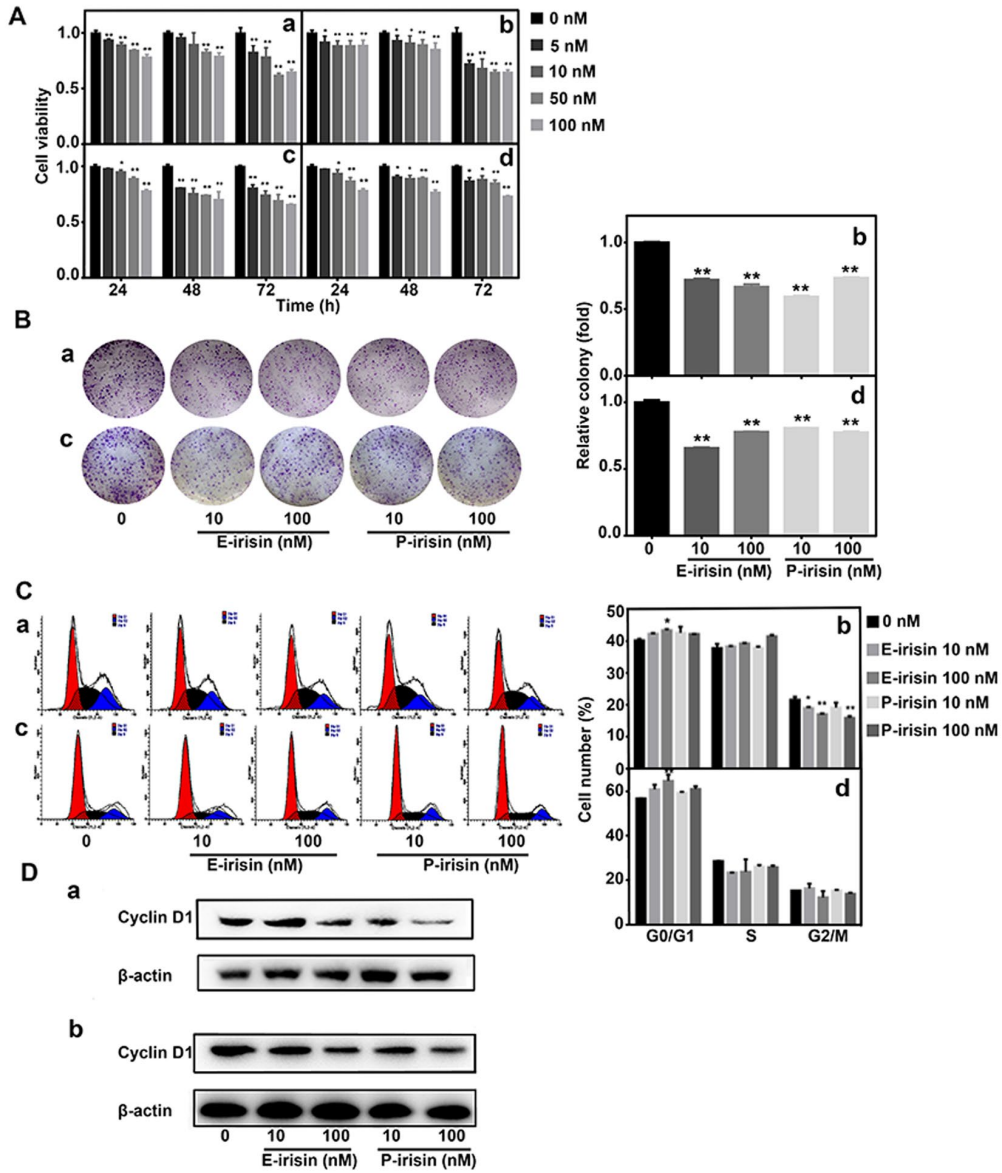
Irisin inhibits the growth of pancreatic cancer cells. (**A**) MIA PaCa-2 (a,b) and Panc03.27 cells (c,d) treated with various concentrations (0, 5, 10, 50, and 100 nM) of E-irisin (a,c) or P-irisin (b,d) for different time points (12, 24, and 48 h). The viability of MIA PaCa-2 and Panc03.27 pancreatic cancer cells was detected using MTT assay. (**B**) Colony formation assay of MIA PaCa-2 (a) and Panc03.27 (c) cells treated with E-irisin or P-irisin, colonies were stained with crystal violet. Quantification of colony formation efficiency in MIA PaCa-2 (b) and Panc03.27 (d) cells. (**C**) Cell cycle distribution analysis. MIA PaCa-2 (a) and Panc03.27 (c) cells were treated with irisin, and cell cycle distribution was evaluated by flow cytometry. Percentages of the cell population of the cell cycle in MIA PaCa-2 (b) and Panc03.27 (d) are shown. (**D**) Western blotting analysis for the expression level of the cell cycle regulatory protein in MIA PaCa-2 (a) and Panc03.27 (b) pancreatic cancer cells, cyclin D1, after treatment with irisin. β-actin was used as the loading control, the original full-length Western blot images are showed in Supplementary Fig. S2. *p < 0.05, **p < 0.01.

The effects of irisin on PC growth were evaluated using the MTT assay. Various concentrations (0, 5, 10, 50, and 100 nM) of E-irisin (as shown in Supplementary Fig. S1, the recombinant E-irisin is expressed and purified successfully) or P-irisin were added into MIA PaCa-2 and Panc03.27 cells in DMEM, respectively, for different time periods (24, 48, and 72 h). As shown in Fig. 2A, MIA PaCa-2 cell viability decreased with an increase in the time of culture and doses of both E-irisin and P-irisin. Panc03.27 cell viability was also significantly reduced by irisin compared with the control group but there was no significant concentration dependence. The cell viability of both the cells was significantly affected in the presence of 10 and 100 nM of irisin at 24 h; thus, these two concentrations were used for the subsequent experiments. Furthermore, the colony formation capacity in MIA PaCa-2 and Panc03.27 cells incubated with E-irisin or P-irisin (0, 10, and 100 nM) was estimated at 14 d after treatment. As shown in Fig. 2B, both E-irisin and P-irisin significantly decreased colony formation capacity of both the cells, respectively compared with the controls. In order to further identify the growth suppressive effect of irisin, the distribution of cell cycles after incubation with irisin for 24 h was examined by flow cytometry. As shown in Fig. 2C, irisin induced cell cycle arrest in G0/G1 phase in MIA PaCa-2 and Panc03.27 cells. When compared with the control group, the percentage of G0/G1 phase in the E-irisin-treated MIA PaCa-2 group and P-irisin-treated MIA PaCa-2 group with high peptide concentrations was increased by 3.1% and 2.1%, respectively. In contrast, the percentage of G0/G1 phase in PANC03.27 cells treated with high concentrations of E-irisin and P-irisin was increased by 7.9% and 4.6%, respectively. Western blot was performed to detect cell cycle-related molecule, cyclin D1. As shown in Fig. 2D, irisin induced the downregulation of cyclin D1 in MIA PaCa-2 and Panc03.27 cells, and β-actin served as loading control. Hence, these results indicated that irisin exhibited an inhibitory effect on the growth of PC cells.

### Irisin inhibits the migration and invasion of PC cells

**Figure 3:**
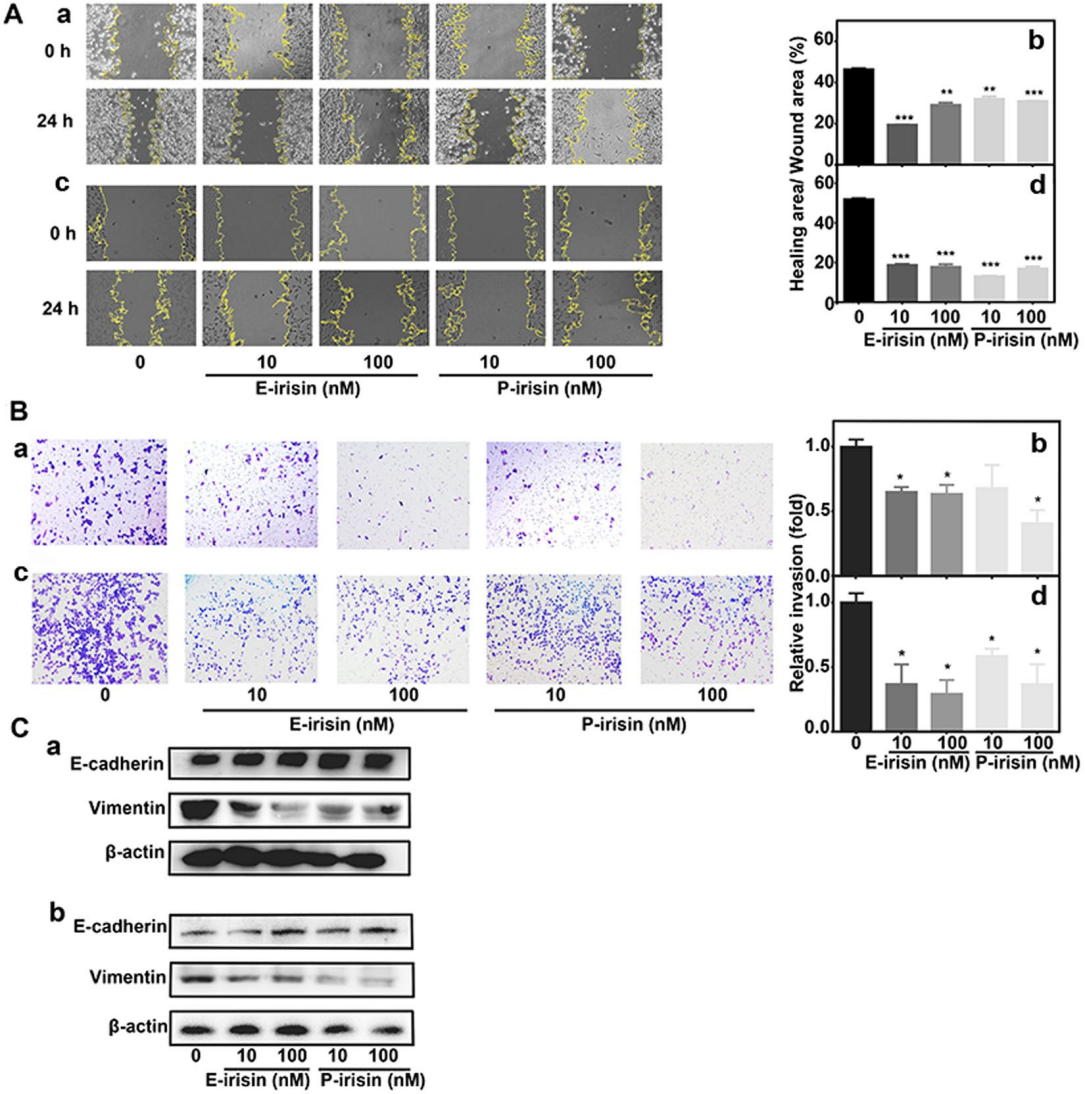
Irisin inhibits migration and invasion of pancreatic cancer cells via inhibition of EMT pathway. (**A**) Images of the wound healing assay in MIA PaCa-2 (a) and Panc03.27 (c) cells at 0 h and 24 h. Quantitative analysis of the wound healing assay by ImageJ in MIA PaCa-2 (b) and Panc03.27 (d) cells. (**B**) Transwell migration and invasion assays of MIA PaCa-2 (a) and Panc03.27 (c) cells after treatment with irisin. Quantitative analysis of the transwell assay in MIA PaCa-2 (b) and Panc03.27 (d) cells. (**C**) Western blotting analysis for the expression level of EMT markers (E-cadherin and vimentin) in MIA PaCa-2 (a) and Panc03.27 (b) cells after treatment with irisin. β-actin was used as the loading control, the original full-length Western blot images are showed in Supplementary Fig. S3. *p < 0.05, **p < 0.01.

The wound healing assay was performed to study the irisin-induced regulation of migration and invasion of PC cells. The MIA PaCa-2 and Panc03.27 cells were treated with E-irisin or P-irisin (0, 10 nM and 100 nM) for 24 h. As shown in Fig. 3A, the healing over the scratch of MIA PaCa-2 and Panc03.27 was decreased after treatment with irisin. Moreover, the invasion ability after adding E-irisin or P-irisin (0, 10, 100 nM) was investigated using the transwell migration and invasion assay. As shown in Fig. 3B, the number of the tested group cells that moved to the lower surface of the transwell chamber membrane was dramatically reduced compared with that of the control cells. The results suggested that both E-irisin and P-irisin decreased cell mobility and invasiveness of MIA PaCa-2 and Panc03.27 cells.

### Irisin inhibits migration and invasion of PC cells via inhibition of epithelial-to-mesenchymal transition pathway

Epithelial-to-mesenchymal transition pathway (EMT) is an important cellular program during tumor migration, invasion, and metastasis. In the process of EMT, epithelial cells lose cell-cell contacts, cell polarity and epithelial markers, particular E-cadherin, while acquiring mesenchymal markers such as vimentin^21^, therefore, loss of E-cadherin expression and acquisition of vimentin are the major features of EMT. To explore the relationships between irisin treatment and PC cell metastasis, Western blot analysis was performed to measure the effect of irisin on EMT marker expression. Various concentrations of E-irisin or P-irisin (0, 10, and 100 nM) were added to in MIA PaCa-2 and Panc03.27 cells, and the cells were cultured for 24 h. Figure 3D shows the expression levels of the epithelial markers^14,15,22^. Among them, E-cadherin was upregulated, whereas vimentin was downregulated in MIA PaCa-2 and Panc03.27 cells compared with those in the control groups, and β-actin served as loading control. Hence, it is apparent that EMT was involved in the irisin-induced inhibition of PC metastasis.

### Irisin-mediated activation of AMPK-mTOR pathway

**Figure 4:**
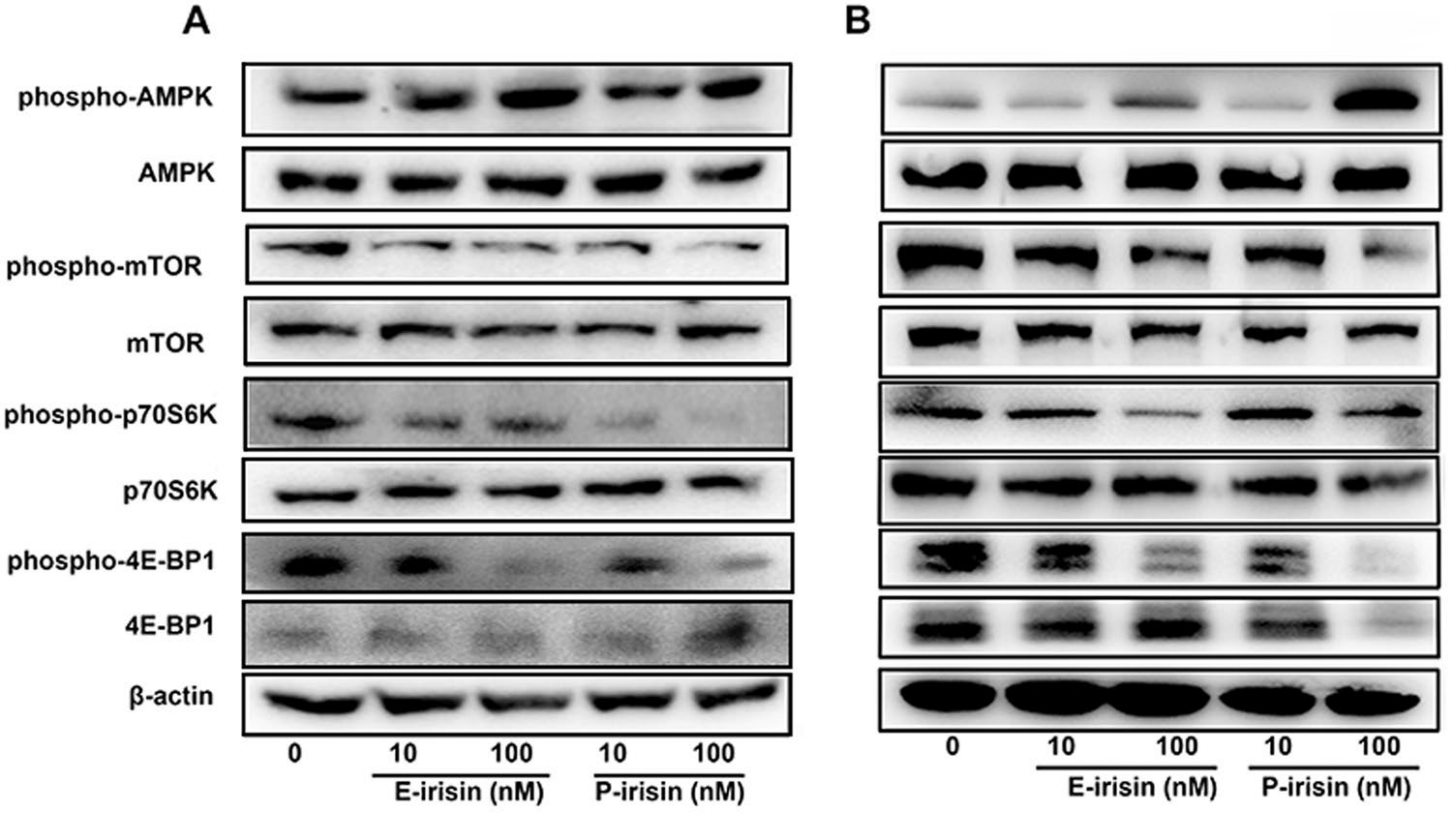
Irisin-mediated activation of the AMPK-mTOR pathway. The phosphorylated (Thr172) and total AMPK, phosphorylated (Ser2448) and total mTOR, phosphorylated (Thr389) and total p70S6 kinase, and phosphorylated (Thr37/46) and total 4E-BP1 proteins of MIA PaCa-2 (**A**) and Panc03.27 (**B**) cells were detected using Western blotting analysis after treatment with E-irisin or P-irisin for 24 h. β-actin was used as the loading control, the original full-length Western blot images are showed in Supplementary Fig. S4.

It is reported that the AMPK-mTOR pathway plays a critical role in cancer cell growth^23^. It is noteworthy that irisin induced AMPK phosphorylation in various cells^24–27^. To investigate the underlying molecular mechanisms involved in the irisin-induced anti-growth effect in PC cells, the influence of irisin on the AMPK-mTOR pathway was examined. The phosphorylation status of AMPK, mTOR, p70S6K, and 4E-BP1 proteins of MIA PaCa-2 and Panc03.27 cells was detected by Western blotting analysis after 24-h treatment with irisin. As shown in Fig. 4, irisin (10 nM and 100 nM) led to significant increase in the phosphorylation of AMPKα at Thr172 and decrease in the phosphorylation of the AMPK pathway downstream signaling molecules including mTOR at ser2448, P70S6K at Thr389, and 4E-BP1 at Thr37/46. β-actin served as loading control.

## Discussion

Although the mortality rate for PC is quite high, the precise causes of PC are still unknown^1,2^. Basen-Engquist *et al*. have shown a link between the incidence of obesity and cancer incidence overall and for selected cancer sites (*e.g*., endometrial, postmenopausal breast, colon, and esophageal adenocarcinoma), concluding that a healthy body weight helps can help people to prevent cancer^12^. Carreras-Torres *et al*. suggested that increase in BMI and fasting insulin were causally associated with an increased risk of PC^6^. Irisin was shown to increase energy expenditure in mice and humans; therefore, it can be considered as a potential treatment option for obesity^7,28^. Serum levels of irisin were significantly lower in breast cancer patients compared to those in normal individuals, thus establishing the relationship between plasma or tissue irisin level and cancer^29^. Irisin has been reported to influence the physiological activity of various cancer cells^13,14,16^. Aydin *et al*. demonstrated that irisin levels were increased in nearly all gastrointestinal cancers, except in liver cancers, by immunohistochemical studies^17^, and reported a significant increase in irisin level in the pancreatic intra- and interlobular ducts of ductal adenocarcinoma compared to normal pancreas. Based on the previous studies, it is remarkable to understand the relationships between pancreatic cancer and irisin.

In this study, the inhibitory effect of irisin against both the pancreatic cell lines (MIA PaCa-2, Panc03.27) was investigated. The MTT and colony formation assay revealed the inhibitory effect of irisin to be concentration-dependent in nature. Flow cytometry study exhibited that irisin could induce cell cycle arrest in G0/G1 phase in MIA PaCa-2 and Panc03.27 cells. It is known that EMT plays an important role in the development of a pancreatic tumor, especially in the progression of invasion and metastasis^21,30,31^. It has been reported that irisin can inhibit EMT in lung cancer^14^ and reverse the IL-6 induced EMT in osteosarcoma cell migration and invasion^15^. Our data demonstrated that irisin can effectively suppress EMT and thus inhibit migration and invasion of MIA PaCa-2 and Panc03.27 cells. As for the two pancreatic cancer cell lines used in the present work, we observed that cell viability of both MIA PaCa-2 and Panc03.27 was reduced by irisin compared with the control group. But the cell viability was decreased more obviously with an increase in the time of culture and doses of both E-irisin and P-irisin in Mia PaCa-2 cell. The percentage of G0/G1 phase in the E-irisin-treated MIA PaCa-2 group and P-irisin-treated MIA PaCa-2 group with high peptide concentrations was increased by 3.1% and 2.1%, respectively. In contrast, the percentage of G0/G1 phase in PANC03.27 cells treated with high concentrations of E-irisin and P-irisin was increased by 7.9% and 4.6%, respectively, thus, irisin has more notable effect on PANC03.27 cells. It is quite clear that effect of irisin on pancreatic cancer cell growth depends on the cell type. It has been reported that the genes, protein level, and cellular molecular mechanisms of MIA PaCa-2 and Panc03.27 are different^32^. Besides, it also suggests that there may be other mechanisms for the influence of irisin on pancreatic cancer cells.

**Figure 5:**
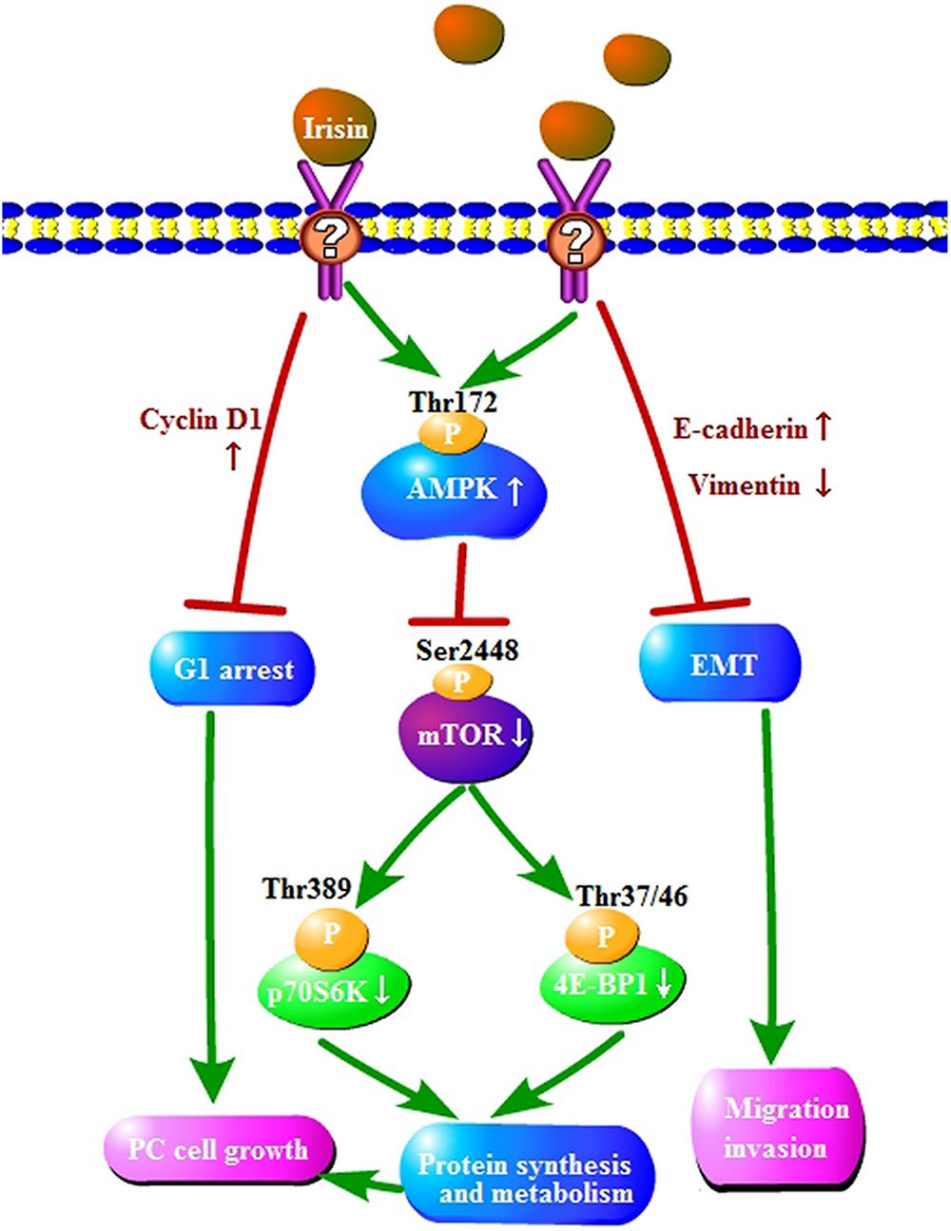
Proposed model of irisin effects on pancreatic cancer. AMPK: adenosine monophosphate-activated protein kinase, P: phosphorylation sites, EMT: epithelial- mesenchymal transition, p70S6 kinase: ribosomal protein S6 kinase, 4E-BP1: eukaryotic translation initiation factor 4E binding protein 1, PC: pancreatic cancer.

Although the mechanism underlying the effect of irisin on pancreatic cancer cells is unclear, AMPK-mTOR pathway was investigated to explain the irisin-induced inhibition of PC growth. AMPK is considered as an energy sensor, and AMPK activity plays important roles in the regulation of energy homeostasis^23,33^. AMPK controls metabolic decisions of cells and has also been confirmed to be involved in various processes of cancer cells^34^. AMPK activation leads to regulation of multiple downstream pathways that are related to the control of cellular proliferation, including inhibition of the mTOR-p70S6K/4E-BP1 pathway, which serves as the major regulator of cell growth and organ size^35,36^. The AMPK pathway is also required for the induction of EMT^37^. AMPK activation can reverse EMT in papillary thyroid cancer cells^38^. Previously, irisin has been reported to be related to the activation of AMPK^25–27,39^. As shown in Fig. 5, the current study demonstrates that irisin can suppress the mTOR-p70S6K/4E-BP1 pathway following the activation of AMPK. Although the receptors of irisin on the membrane of cardiomyoblasts^18^ and preadipocytes^19^ have been detected, it is noteworthy to detect irisin-specific receptors on the membrane of pancreatic cells. In addition, the previous research has shown that irisin consists of an N-terminal fibronectin III (FNIII)-like domain attached to a flexible C-terminal tail which forms a continuous intersubunit β-sheet dimer, it also confirmed that irisin is a dimer and that dimerization is unaffected by glycosylation^20^. In this study, human recombinant nonglycosylated P-irisin in *E. coli* and human recombinant glycosylated E-irisin in *P. pastoris* were expressed and purified; however, as there is no obvious difference between the two irisin, the anticancer activity of irisin remains unaffected by glycosylation, which is consistent with the previous report that dimerization of irisin is unaffected by glycosylation^20^.

In conclusion, our data demonstrated that irisin suppresses the migration, and invasion of MIA PaCa-2 and Panc03.27 cells by inhibiting EMT. We demonstrated that irisin activates the AMPK-mTOR signaling pathway, which may play a critical role in irisin-induced inhibition of pancreatic cancer cell growth (Fig. 5). We consider that irisin may be employed as a potential therapeutic candidate for the treatment of pancreatic cancer in clinical practices.

## Material and Methods

### Expression and purification of human recombinant glycosylated E-irisin

Human His-irisin cDNA was designed and synthesized in a pPIC9k plasmid *by Sangon Biotech* (Shanghai, China). The plasmid pPIC9K-His-irisin DNA was linearized by restriction digestion with 0.3 U/µl SacI (TaKaRa, Kusatsu, Japan). Typically, 2 μg of SacI-linearized was mixed with 80 μl of competent GS115 cells. The cell mixture was then transferred to an ice-cold 0.2 cm electroporation cuvette (Bio-Rad Laboratories Inc, Philadelphia, PA, USA) and kept on ice for 5 min. Subsequently, the cell mixture was pulsed at 2000 V, 25 μF of capacitance, and 200 Ω of resistance for 5 ms using a Gene Pulser Xcell apparatus (Bio-Rad, PA, USA). The transformed cells were painted on MD (1.34% yeast nitrogen base, 4 × 10^−5^% biotin, 2% dextrose, and 2% agar) plates and cultured at 30 °C for 4 d. The protein expressing strains were obtained using G418 selection and cultured in 25 ml of *buffered glycerol-complex medium* [BMGY; 1% yeast extract, 2% peptone, 100 mM potassium phosphate (pH 6.0), 1.34% yeast nitrogen base, 4 × 10^−5^% biotin, and 1% glycerol] at 30 °C for 24 h. After the OD600 value reached three to four, cells were harvested by centrifugation and resuspended in 30 ml of *buffered* methanol-complex *medium (*BMMY) [1% yeast extract, 2% peptone, 100 mM potassium phosphate (pH 6.0), 1.34% yeast nitrogen base, 4 × 10^−5^% biotin, and 0.5% methanol] to an OD600 of 1.0. Subsequently, the cells were incubated at 30 °C for 4 d under shaking at 250 rpm with 5% methanol added to the medium daily. For expression of human recombinant glycosylated E-irisin, the induced supernatant was filtered through a 0.45μm filter. The supernatant was concentrated by adding ammonium sulfate to a final saturation of 40%. The precipitated proteins were collected by centrifugation at 20,000 × *g* for 20 min and redissolved in binding buffer (50 mM Tris-HCl, pH 8.0) overnight. The supernatant containing His-irisin was incubated with Ni-NTA agarose for 1 h in the column. The Ni-affinity column was washed with washing buffer (50 mM Tris-HCl, 70 mM imidazole, pH 8.0), and His-irisin was eluted with elution buffer (50 mM Tris-HCL 300 mM imidazole, pH 8.0). The target protein was verified by Western blotting using anti-irisin antibody (Phoenix Pharmaceuticals, USA) and stained with periodic acid-Schiff to confirm glycosylation. Protein concentrations were determined using bicinchoninic acid (BCA) Protein Assay Kit from *Thermo* Fisher Scientific (*MA*, USA). Human recombinant nonglycosylated P-irisin was expressed and purified as previously described^40^.

### Reagents and antibodies

Anti-Irisin (Human, Rat, Mouse, Canine specific) antibody was purchased from Phoenix Pharmaceuticals (CA, USA). Fluorescein isothiocyanate (FITC)-anti-rabbit IgG antibody was from BIOSS (Beijing, China). MTT was purchased from Sigma -Aldrich (MO, USA). Honchest33258 was purchased from Solarbio (Beijing, China). Anti-E-cadherin, anti-vimentin, and anti-cyclin D1 rabbit pAb were purchased from Wanleibio (Shenyang, China). Anti-total and anti-phosphorylated (Thr172) AMPK, anti-total and anti-phosphorylated (Ser2448) mTOR, anti-total and anti-phosphorylated (Thr389) p70S6 kinase (ribosomal protein S6 kinase), anti-total and anti-phosphorylated (Thr37/46) 4E-BP1 (eukaryotic translation initiation factor 4E binding protein 1), anti-beta actin rabbit antibodies, and anti-rabbit horseradish peroxidase (HRP)-conjugated IgG antibodies were obtained from Cell Signaling Technology (MA, USA). The enhanced chemiluminescence (ECL) detection reagent was from Millipore (CA, USA).

### Cell lines

MIA PaCa-2 and Panc03.27 cells were purchased from ATCC (Manassas, USA) and cultured in Dulbecco’s Modified Eagle Medium (DMEM) (Gibco, NY, USA) supplemented with 10% fetal bovine serum (FBS) (Kang Yuan Biology, Tianjin, China), 1% penicillin (100 U/mL), and streptomycin (100 ìg/mL) (Gibco) at 5% CO_2_, 37 °C under a humidified atmosphere.

### Immunofluorescent staining

Immunofluorescent staining was used to detect the presence of irisin receptors on the membrane of PC cells. MIA PaCa-2 and Panc03.27 cells were inoculated into confocal dishes (NEST Biological Technology Co., Ltd., Shanghai, China) at 2 × 10^5^ cells density with DMEM (10% FBS). The cells were incubated with or without irisin for 1 h. After washing with PBS thrice, the cells were fixed in pre-cooled 75% ethyl alcohol for 1 h at −20 °C and washed thrice for 5 min with 1 mL of 1× PBS. Subsequently, the cells were washed thrice for 5 min with 1 mL 1× PBS, incubated with anti-irisin rabbit antibody (1:1000), and washed thrice for 5 min with 1 mL 1× PBS. The cells were incubated with FITC-anti-rabbit IgG antibody (1:5000) for 1 h and subsequently stained with Honchest33258. Finally, the cells were washed and observed under confocal fluorescence microscope.

### Cell MTT assay

The MTT assay was performed to evaluate cell growth. Briefly, MIA PaCa-2 and Panc 03.27 cells were seeded in 96-well plates (1 × 10^4^ cells/well) in DMEM (10% FBS) and allowed to attach overnight. The cells were treated with various concentrations (0, 5, 10, 50, and 100 nM) of E-irisin or P-irisin in DMEM, respectively for different time periods (24, 48, and 72 h). Subsequently, 200 μl of dimethyl sulphoxide was added to each well to dissolve the formazan crystals. The OD was measured at 570 nm using a multimode reader Infinite F200 Pro (TECAN, Switzerland). The cell viability was calculated for each well as A570 treated cells/A570 control cells × 100%.

### Colony formation assay

For colony formation assay, MIA PaCa-2 and Panc03.27 cells were seeded in 6-well plates at a density of 500 cells per well and incubated overnight in DMEM (10% FBS). Subsequently, the cells were treated with different concentrations (0, 10, and 100 nM) of E-irisin or P-irisin. After a 2-week treatment period, the cells were washed with 1× PBS and fixed in methanol for 15 min. Subsequently, after washing with PBS, the cells were stained with 0.2% crystal violet (dissolved in 10% acetic acid), washed twice with 1× PBS, and observed and photographed under Olympus microscope (Tokyo, Japan). The absorbance values were determined at 595 nm.

### Flow cytometry analysis

For cell cycle analysis, MIA PaCa-2 and Panc03.27 cells were seeded in 6-well plates (3 × 10^5^ cells/well) overnight. The cells were synchronized by serum starvation and treated with various concentrations (0, 10, and 100 nM) of E-irisin or P-irisin for 24 h. Subsequently, the cells were washed with 1× PBS and fixed in 75% ethanol for 1 h at −20 °C. Then the cells were washed and centrifuged twice at 1000 rpm for 5 min, After wash, the collected cells were resuspended in 500 ìl 1× PBS with RNase A and propidium iodide for 30 min at room temperature. The cells were then analyzed by flow cytometry (BD Bioscience, NY, USA).

### Wound healing assay

MIA PaCa-2 and Panc03.27 cells were plated in 6-well plates (3 × 10^5^ cells/well) in DMEM containing 10% FBS and allowed to grow until confluency. Next, a wound was made by scratching the monolayer cells with a sterile 200-ìl pipette tip. After washing with 1× PBS, cells were treated with irisin as described in the flow cytometry analysis and photographed under Olympus microscope. The remaining wound area was measured by ImageJ software (National Institutes of Health, USA) at 0 h and 24 h after the scratch to evaluate the migration ability of the cells.

### Transwell migration assay

The transwell migration assay was performed to evaluate the effect of irisin on cell migration and invasion. MIA PaCa-2 and Panc03.27 cells were plated in 6-well plates (2 × 10^5^ cells/well) with DMEM containing 10% FBS overnight and then treated with various concentrations (0, 10, and 100 nM) of E-irisin or P-irisin without serum for 24 h. The treated cells were collected and loaded into the upper chambers of the transwell chambers with serum-free DMEM containing 1% BSA. DMEM containing 10% FBS was added to the lower chambers. After incubation for 24 h, the lower side of the upper chamber membranes was fixed with 75% cold ethanol, stained with 0.2% crystal violet, and washed with 1× PBS twice. Images were taken under Olympus microscope.

### Western blot analysis

Total proteins were separately extracted from MIA PaCa-2 and Panc03.27 cells, which were treated as described in the transwell migration assay (a pre-experiment revealed irisin-induced AMPK phosphorylation persists for at least 24 h in MIA PaCa-2 cells, unpublished data). The cells were washed with pre-cooled 1× PBS and solubilized. After incubation in ice for 30 min, the lysates were centrifuged at 12000 rpm for 20 min at 4 °C. Protein concentration in the supernatant was measured using BCA Protein Assay Kit. The same amount of protein was loaded into each well of 10% sodium dodecyl sulfate-polyacrylamide gel electrophoresis (SDS-PAGE) and electrotransferred to PVDF membranes (Millipore, CA, USA). The PVDF membranes were blocked with 5% nonfat dry milk (Solarbio, Beijing, China) in TBST (10 mM Tris-HCl, 150 mM NaCl, and 2% Tween) for 1 h at room temperature and incubated with primary antibodies (1:1000) at 4 °C overnight. Subsequently, PVDF membranes were washed with TBST thrice for 5 min and incubated with (HRP)-conjugated secondary antibodies for 1 h. Subsequently, the PVDF membranes were washed with TBST. Then the blots were visualized using an enhanced chemiluminescence substrate and detected using the Tanon 2500 chemiluminescence imaging system (Shanghai, China).

### Statistical analysis

The data are presented as the mean ± standard deviation (SD). The statistical significance of differences between different experimental groups and the control group were analyzed using student’s t-tests GraphPad Prism 5.0 software (GraphPad Software, San Diego, CA, USA), p < 0.05 was considered to be statistically significant.

## Electronic supplementary material

Supplementary file

## Data Availability

All data generated or analyzed during this study are included in this published article (and its Supplementary Information files).
