# Supplementary material for: Irisin inhibits pancreatic cancer cell growth via the AMPK-mTOR pathway
Source: Sci Rep. 2018 Oct 15;8:15247. doi: 10.1038/s41598-018-33229-w (PMC6189061; doi:10.1038/s41598-018-33229-w)
Supplement: Supplementary file 1 — Supplementary file [file 41598_2018_33229_MOESM1_ESM.pdf]

Irisin inhibits pancreatic cancer cell growth via the AMPK-mTOR pathway

Jiayu Liu [1,2], Nannan Song [1,2], Yibing Huang [1, 2], Yuxin Chen [1,2*]

1 Key Laboratory for Molecular Enzymology and Engineering of the Ministry of Education, Jilin University, 2699 Qianjin Street, Changchun, 130012, China

2 School of Life Sciences, Jilin University, Changchun, 130012, China

***Corresponding Author:** Yuxin Chen, Key Laboratory for Molecular Enzymology and Engineering of the Ministry of Education, Jilin University, 2699 Qianjin St., Changchun, Jilin, P. R. China 130012. Tel.: +86-431-85155220 Fax: +86-431-85155200 Email: chen_yuxin@jlu.edu.cn.

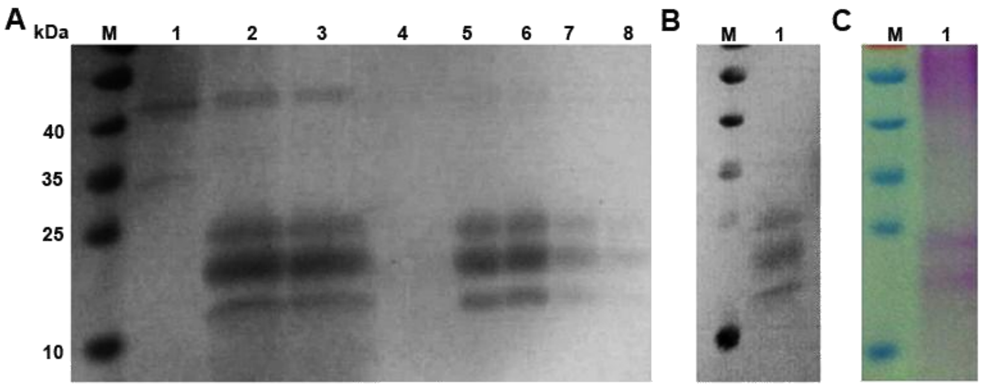

Supplementary Figure S1: Expression and purification of human recombinant glycosylated E-irisin A.SDS–PAGE analysis of expression of recombinant E-irisin in GS115-pPIC9k-E-irisin *Pichia pastoris* transformants and purification of E-irisin from *Pichia pastoris* transformants culture. Lanes: M protein marker. 1 the cultured supernatant of *Pichia pastoris* transformants before induction (negative control). 2 the cultured supernatant of *Pichia pastoris* transformants after induced with 5% methanol, (before passing Ni affinity column). 3 sample after passing Ni affinity column. 4 the washout of the Ni affinity column with 70 mM imidazole. 5-8 the washout of the Ni affinity column with 300 mM imidazole. **B:** The target E-irisin protein was verified by western blotting using anti-irisin antibody: Lanes: M protein marker. 1 the purified E-irisin. Three protein bands with molecular mass about 17, 20 and 25 kDa. **C:** The purified E-irisin stained by periodic acid-Schiff. Lanes: M protein marker. 1 the purified E-irisin. Two higher molecular weights (25 and 20 kDa) of the secreted protein were dyed purple by acid-Schiff.

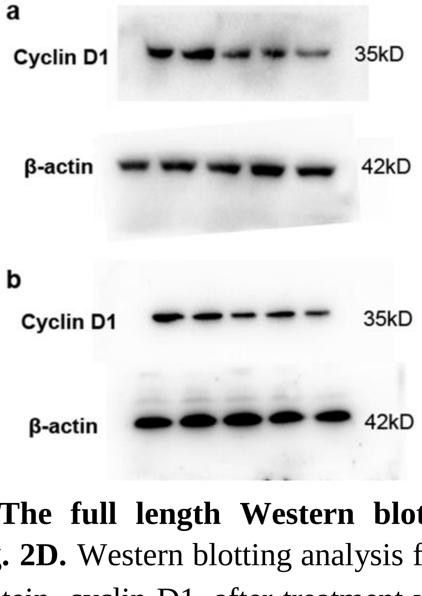

Supplementary Figure S2: The full length Western blotting images for the cropped images shown in Fig. 2D. Western blotting analysis for the expression level of the cell cycle regulatory protein, cyclin D1, after treatment with irisin. β-actin was used as the loading control. MIA PaCa-2 **(a)** and Panc03.27 **(b)**.

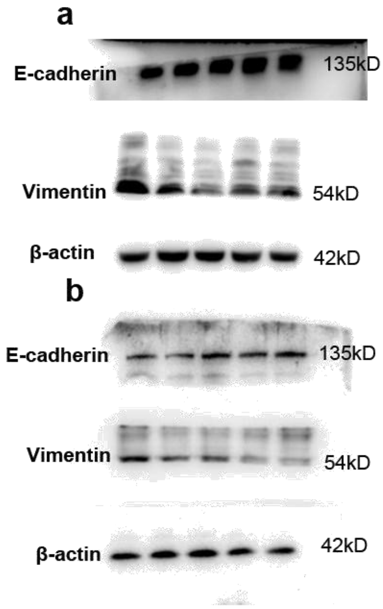

Supplementary Figure S3: The full length Western blotting images for the cropped images shown in Fig. 3C. Western blotting analysis for the expression level of EMT markers (E-cadherin and vimentin) in MIA PaCa-2 **(a)** and Panc03.27 **(b)** cells after treatment with irisin. β-actin was used as the loading control.

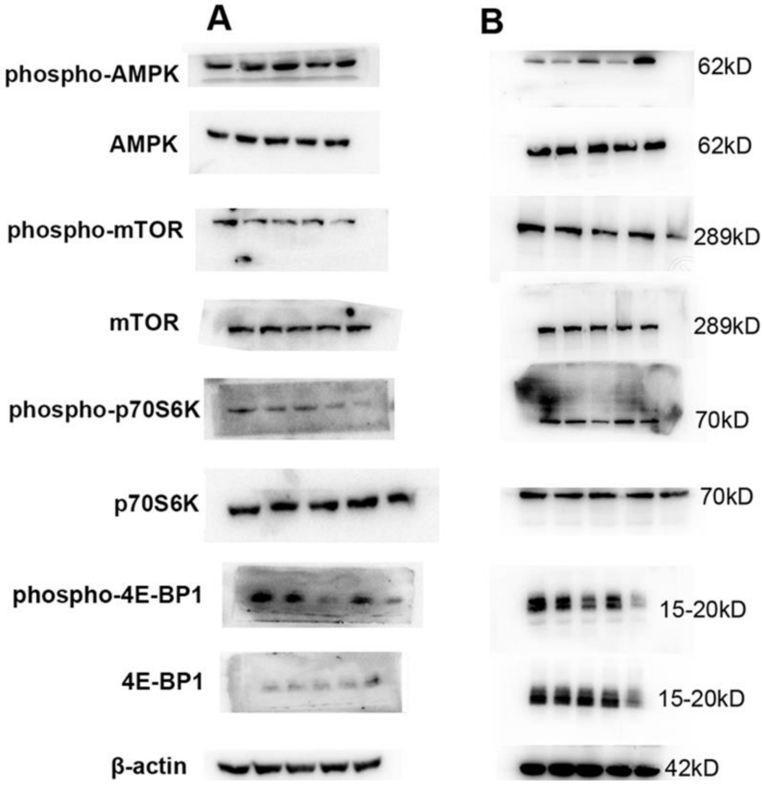

Supplementary Figure S4: The full length Western blotting images for the cropped images shown in Fig. 4. The phosphorylated (Thr172) and total AMPK, phosphorylat-

ed (Ser2448) and total mTOR, phosphorylated (Thr389) and total p70S6 kinase, and phosphorylated (Thr37/46) and total 4E-BP1 proteins of MIA PaCa-2 **(A)** and Panc03.27 **(B)** cells were detected using Western blotting analysis after treatment with E-irisin or P-irisin for 24 h. β-actin was used as the loading control.
